# The Relationships Between Hardiness and Life Satisfaction or Expectation of Korean Multicultural Children: Focused on Mediating Effect of Acculturative Stress Moderated by Resilience

**DOI:** 10.3389/fpsyg.2021.663134

**Published:** 2021-10-18

**Authors:** Bok-Hee Kim, Kyung-Hyun Suh

**Affiliations:** Department of Counseling, Sahmyook University, Seoul, South Korea

**Keywords:** acculturative stress, hardiness, resilience, life satisfaction, expectation, multicultural children

## Abstract

This study identified the relationship between hardiness and life satisfaction or expectation of multicultural Korean children, and examined the mediating models of acculturative stress moderated by resilience on hardiness and life satisfaction or life expectation. The participants in the study were 201 male and female children from Korean multicultural families. PROCESS Macro 3.5 Model 14 was used for the analysis of the moderated mediating effects. The results revealed that hardiness and resilience were positively correlated with life satisfaction and life expectation, whereas acculturative stress was negatively correlated with life satisfaction and life expectation of multicultural children. In a moderated mediating model for life satisfaction, there was no interaction effect of acculturative stress and resilience, but a conditionally indirect effect of acculturative stress was only significant in groups with low resilience. In a moderated mediating model for life expectation, there was a significant interaction effect of acculturative stress and resilience, and a conditionally indirect effect of acculturative stress was only significant in groups with low resilience and with very high resilience. These findings suggest that only when multicultural children have low resilience, the mediating effect of acculturative stress is significant in relations of hardiness and life satisfaction, as well as hardiness and life expectation. In particular, resilience moderates the influence of acculturative stress on life expectations.

## Background

According to a report by Statistics Korea (2020), the Korean National Statistical Office, six out of every 100 babies born in Korea in 2019 were from multicultural families. Along with the decrease in the total birth rate decreased in Korea, the number of 17,939 births from 2019–2020, however, the ratio of multicultural births in relation to all births is expected to increase, as there has been an increase in the number of multicultural marriages natioanlly (Statistics Korea, 2020). The report revealed that multicultural families in Korea mostly involve married immigrant women. Although the proportion of multicultural children is increasing, they are likely to experience stress in Korean society because of their minority status. Considering the expected growth of this population, the stress experienced by multicultural children in Korean society is an increasingly important social issue (e.g., Kim, 2012).

A large body of research shows that stress threatens mental health (Dohrenwend and Dohrenwend, 1974), and reduces measures of subjective well-being, such as including life satisfaction (Ng et al., 2009). Acculturative stress can also negatively affect the mental health of adolescents (Sirin et al., 2013, 2019). In particular, if people in the mainstream society have negative prejudices regarding individuals' cultural origins, adolescents of multicultural background are likely to experience psychological difficulties or mental health problems (Goforth et al., 2016). In a recent study by Bae (2020), the more adolescents from Korean multicultural families who experienced acculturative stress, reported poorer psychological well-being. In contrast, Yoon et al. (2008) found that acculturation could increase subjective well-being through social connection in the mainstream society. Another study (Yi, 2017) established that acculturative stress in international students in the United States was positively related to their life satisfaction as well as positive affect. However, studies have not investigated whether the acculturative stress in multicultural children can affect the expectation or the hope for their future lives. In order to develop a clearer picture of how acculturative stress affects multicultural children in the Korean context, this study explores the relationship between acculturative stress and life satisfaction in this population.

In childhood and adolescence, an individuals' expectation for the future may be more important than a feeling of satisfaction with their present lives. Kim et al. (2009) argued that expectations for their future are more important than life satisfaction as a factor for the subjective well-being of young people. Eckersley et al. (2005) also emphasized that young people are very focused on determinant about their future lives because they look forward to and are motivated in their lives, even if they are not satisfied with the present. In another study (Suh, 2012), adolescents reported lower level of subjective well-being in terms of life satisfaction and negative/positive affect than adults, but the relationship between life satisfaction and feelings of happiness in adolescents differed according to their expectation for their future. Additionally a study of Korean elementary school students (Cho, 2011) found that hope for their future might promote their psychological well-being in childhood. As some studies (Thompson et al., 2012; Kim and Suh, 2016) revealed that stress in childhood or adolescence might lower individuals' expectations for the future, this study assumes that acculturative stress could also lower the life expectations of multicultural children.

In this study, it is assumed that multicultural children with low hardiness would experience more acculturative stress. Hardiness was first introduced by Kobasa (1979) as a personality trait that prevents a person from experiencing stress in circumstances where there are stressors. Kobasa et al. (1982) viewed hardiness as a resistant resource during stressful conditions and as a dispositional trait with three general factors: commitment, control, and challenge. Research (Gill and Harris, 1991; Klag and Bradley, 2004) demonstrated that an individual's hardiness of people helps them maintain their physical and mental health by making them less likely to experience negative emotions and stress. This finding suggests that hardiness has a buffering effect against the negative outcomes of stress. Thus, although the hardiness of multicultural children is yet to be studied, we assumed that multicultural children who are vulnerable to prejudice and discrimination in Korean society will experience less acculturative stress if their hardiness is high.

Some researchers (Hystad et al., 2009) have been interested in the moderating effect of hardiness on the relationship between stress and mental health. However, this study tries to examine, particularly, how the hardy personality of multicultural children affects their experiences of less acculturative stress, as well as their subsequent life satisfaction and expectation for their future lives. Since hardy personality is not a character trait or temperament (Kobasa et al., 1982), the effect of hardiness on acculturative stress, rather than the effect of acculturative stress on hardiness is assume. Bartone (2007) described hardiness as a dispositional resilience. Thus, we consider hardiness as temperament in this study, whereas resilience is an ability that can be developed and acquired.

Given the relationship between hardiness and acculturative stress, we assumed that the resilience of multicultural children could also moderate the relationship between acculturative stress and their life satisfaction or life expectation. Other mediating or moderating variables have been examined previously. For example, Yi (2017) found that an individual's perception of their meaning of life mediated the acculturative stress and the subjective well-being, but this mediating effect was moderated by coping with the strategies of acceptance, reframing, and striving. According to yet another study (Serafica et al., 2019), acculturation did not significantly influence the mental health of older Filipino immigrants in the United States; however, resilience and acculturative stress had significant effects on their mental health.

Pinquart (2009) found moderating effects of resilience on the daily hassles or stressors and psychological distress or negative stress responses of adolescents. A Study by Choi (2017) showed that resilience moderated on the association between stress and quality of life or well-being. In a study by Kim and Koh (2016), found that resilience also moderated the relationship between stress and traumatic childhood experiences that affected the life satisfaction of college students. Through a critical systematic review, Singh and Gujral (2018) concluded that resilience has a positive effect on reducing stressors and moderating the negative effects of stresses. Although it has not yet been studied, because resilience is most widely known as a moderator of stress (Stanley et al., 2018), this study predicted that resilience might play such a role in the relationship between the acculturative stress and life satisfaction or life expectations in multicultural children.

**Figure 1:**
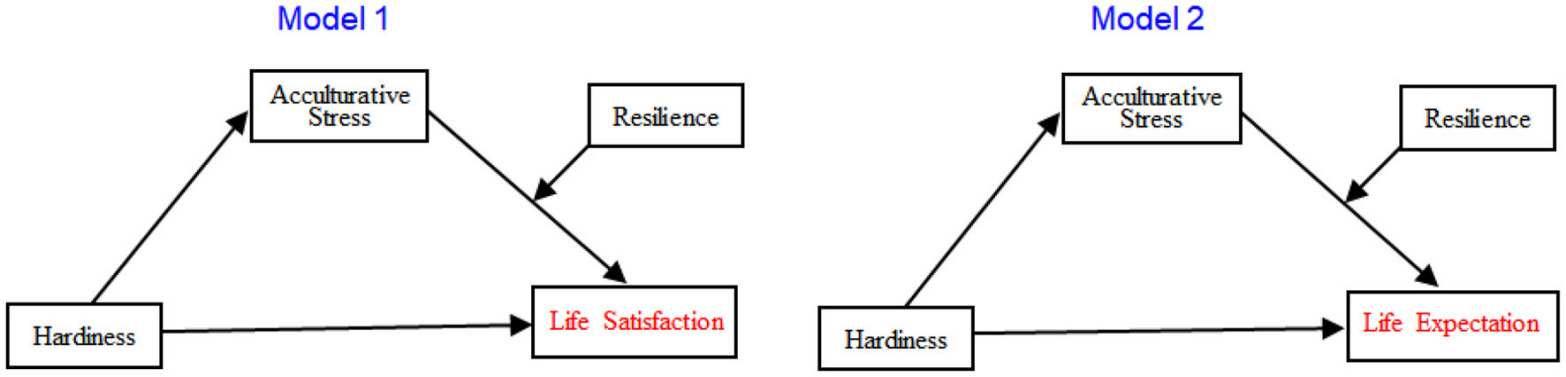
Moderated mediation models of this study.

This study aims to explore the relationship between hardiness and life satisfaction or expectation of children from Korean multicultural families, and examine the moderated mediating effect by resilience through acculturative stress on this relationships between hardiness and life satisfaction or expectation. To achieve this aim, the following hypotheses were raised. First, there are relationships among hardiness, acculturative stress, resilience, and life satisfaction or expectation of children from Korean multicultural families. Second, resilience has a moderating effect on the mediating role of acculturative stress on the relationship between hardiness and life satisfaction (Model 1). Third, resilience has a moderating effect on the mediating role of acculturative stress on the relationship between hardiness and life expectation (Model 2). Two hypothesized models were presented in Figure 1.

## Methods

### Participants

The participants in the study were 201 male and female multicultural children in J County, Gangwon-do, Korea. The criteria for the inclusion were as follows. The first was children from families became multicultural because their mothers, who were foreigners, married Koreans. The second was children, at least in the fifth grade, were able to understand and respond to the questionnaires. To meet the second criterion, children diagnosed with intellectual disability, attention deficit hyperactivity disorder (ADHD), and other mental disorders were excluded. Thirty-three children who did not meet these criteria or responded incompletely were excluded from the analysis.

The final sample included 90 males (44.8%) and 111 females (55.2%) children (Table 1). Their average age was 11.86 (*SD* = 1.01) years. Seventy-seven (38.3%) were in the fifth grade, 72 (35.8%) were in the sixth grade, 29 (14.4%) were in the seventh grade, and 23 (11.5%) were in the eighth grade. The average number of siblings was 1.13 (*SD* = 0.65), with the majority of participants (60.7%) having one sibling. Eighty-nine (44.3%) participants reported living with their grandfathers. The participants' mothers' native countries were the Philippines (55), Cambodia (51), Vietnam (44), China (14), Nepal (13), Japan (6), Thailand (5), Uzbekistan (6), Russia (2), and Canada (2).

**Table 1:** Demographic profile of participants (*N* = 201).

**Variables**		**Frequency**	**Percent (%)**
Gender	Male	90	44.8
	Female	111	30.7
Grade	5th	77	38.3
	6th	72	35.8
	7th	29	14.4
	8^th^	23	11.5
Number of sibling(s)	0	28	13.9
	1	122	60.7
	2	48	23.9
	3	3	1.5
Living with grandfather?	Yes	89	44.3
	No	112	55.7
Living with grandmother?	Yes	150	74.6
	No	51	25.4

## Measures

### Hardiness

In the absence of validated scales to measure the hardiness of children, the hardiness of participants was measured with items used in Hwang (2011) study, which was modified for use in children from items by Kobasa et al. (1982) and Bartone et al. (1989). The scale used by Hwang (2011) consisted of 45 items, but in this study the items were shortened and modified, as in the Bartone's Short Hardiness Scale (Bartone, 1995), in order to make them easier for the children to understand. Therefore, there were no reverse items. As Kobasa et al. (1982) had conceptualized, there were three subscales: commitment, control, and challenge. Some examples of the items are, “Almost every day my life is really fun and interesting (commitment),” “By working hard, I can always achieve my goals (control),” “I like to change my daily life (challenge).” The scale used in this study was composed of 12 items, with the four items from each of the three sub-scales. For the purposes of this study, only the total hardiness was only used in the analysis without classifying them as subfactors. Each item was rated on a 4-point Likert scale ranging from 1 (*absolutely untrue*) to 4 (*absolutely true*). Participants' total scores ranged from 14 to 56. The internal consistency (Cronbach's α) of 12 items in this study was 0.92.

### Acculturative Stress

Acculturative stress of the participants was measured with the Korean version of the societal, attitudinal, familial, and environmental acculturative stress scale for children (K-SAFE-C) which was validated by Un (2010) for Korean children. The SAFE-C was originally developed by Chavez et al. (1997) and it consists of three subscales and 36 items. However, K-SAFE-C consists of 20 items and two subfactors: process-oriented stress (14 items) and perceived discrimination (6 items). For purpose of this study, only the total score was used in the analysis. Some examples of the items are, “I think my friends don't play with me because my mom or dad is a foreigner,” and “I feel uncomfortable in Korea.” Each item was rated on a 5-point Likert scale ranging from 1 (*not at all*) to 5 (*always*). Participants total scores ranged from 31 to 88. The internal consistency (Cronbach's α) was 0.89 for this study.

### Resilience

To measure participants' resilience, we used the Measure of Resilience that was developed by Shin et al. (2009), which was validated for Korean adolescents. This scale consists of 27 items and three subscales that measure controllability, sociability, and positivity. Each subscale has nine items and is divided into three sub-actors. For the purpose of this study, only was used in the analysis for this study. Some examples of the items are, “I can control my emotions well when something difficult happens (controllability),” “I can see how people feel by looking at their facial expressions (sociability),” “I believe everything will be fine even if something difficult happens (positivity).” Participants rated items from 1 (*not at all*) to 5 (*always*), and participants total scores ranged from 38 to 129. The internal consistency (Cronbach's α) of these items in this study was 0.95.

### Life Satisfaction

The participant's levels of life satisfaction were measured with the Satisfaction with Life Scale (SWLS) developed by Diener et al. (1985). The SWLS is an instrument designed to measure global cognitive judgments of the lives of people. Life satisfaction is distinguished from affective appraisal in that it is more cognitively than emotionally driven. Life satisfaction can be assessed specifically to a particular domain of life, or globally. The SWLS consists of five items which participants rated from 1 (*strongly disagree*) to 7 (*strongly agree*). Total scores were ranged from 7 to 35. In this study, internal consistency (Cronbach's α) was found to be 0.90 for five items.

### Life Expectation

The life satisfaction expectancy motivation scale (LEMS), developed by Kim (2007) to measure the life expectation of multicultural children, was used for this study. It includes modified items from SWLS (Diener et al., 1985). Some examples include, “In most ways my life is close to my ideal,” which was modified to “In most ways my life will be close to my ideal in future.” This scale also consists of five items which participants rated from 1 (*strongly disagree*) to 7 (*strongly agree*). Total scores also ranged from 7 to 35. The internal consistency (Cronbach's α) of these items in this study was found to be 0.96.

## Procedure

This study was approved by the Institutional Review Board before conducting the study (approval number: 2-1040781-A-N-012020015HR). For this study, data were collected by researchers with written informed consent from parents of the participants. From June 1, 2020, to July 15, 2020, data were collected in compliance with the guidelines of the Ministry of Education for the prevention of COVID-19. The researchers gave the participants and their guardians disclosed research information to facilitate their understanding of this study and to encourage their cooperation. The participants were informed that they could withdraw at any time while responding to the survey. Each child spent an average of 25 min for completing the questionnaire.

## Statistical Analysis

The G^*^Power 3.1.9 indicated that a minimum sample size of 176 was required to reach a statistical conclusion based on the number of predictors and moderators, a significance level of 0.05, power of 0.95, and effect size of 0.10. IBM SPSS Statistics for Windows 25.0 and PROCESS Macro 3.5 were used for the statistical analyses of this study. Skewness and kurtosis of the data for parametric statistical analyses were checked. Pearson-Product Moment correlational analysis was performed using SPSS, and the analyses of moderated mediating effect were performed with PROCESS Macro 3.5 Model 14 (Hayes, 2018). Finally, bootstrapping using 5,000 resamples with a 95% confidence interval was used to analyze the significance of moderated mediating models. None of the collected demographic profiles met the condition of the confounding variable, so these models were not adjusted by covariates. Additionally, resilience and acculturative stress were mean centered.

## Results

### The Relationship Between Variables Involved in Life Satisfaction and Expectation

Table 2 presents the results of the correlational analysis of hardiness, acculturative stress, resilience, life satisfaction, and life expectation in multicultural children. None of the skewness and kurtosis absolute values exceeded 1.5, thus indicating that the variances of all variables were close to a normal distribution for conducting parametric statistical analyses.

**Table 2:** Correlational matrix of hardiness, acculturative stress, resilience, life satisfaction, and life expectation (*N* = 201).

**Variables**	**1**	**2**	**3**	**4**	**5**
1. Hardiness	1				
2. Acculturative stress	−0.183^**^	1			
3. Resilience	0.556^***^	−0.247^***^	1		
4. Life satisfaction	0.207^**^	−0.218^***^	0.333^***^	1	
5. Life expectation	0.529^***^	−0.256^***^	0.634^***^	0.202^**^	1
*M*	34.48	55.78	85.13	14.39	27.72
*SD*	7.42	11.09	17.28	6.05	7.10
Skewness	−0.13	0.22	−0.21	0.63	−1.27
Kurtosis	−0.05	−0.25	−0.65	0.27	1.35

** *p < 0.01*,

*** *p < 0.001*.

The correlation analysis revealed that hardiness was positively correlated with life satisfaction (*r* = 0.207, *p* < 0.01) and life expectation (*r* = 0.529, *p* < 0.001), while acculturative stress negatively correlated with life satisfaction (*r* = −0.218, *p* < 0.001) and life expectation, *r* = −0.256, *p* < 0.001. Resilience was also positively correlated with life satisfaction (*r* = 0.333, *p* < 0.001) and life expectation (*r* = 0.634, *p* < 0.001). Additionally, life satisfaction was positively correlated with life expectation (*r* = 0.202, *p* < 0.01). This result means that these two variables shared only about 4.1% of the variation.

Hardiness was negatively correlated with acculturative stress (*r* = −0.183, *p* < 0.01), whereas it positively correlated with resilience (*r* = 0.556, *p* < 0.001). In addition, resilience negatively correlated with acculturative stress (*r* = −0.247, *p* < 0.001).

### Verification of the Moderated Mediation Model for the Life Satisfaction

This study examined a moderating mediating effect of resilience through acculturative stress on the hardiness and life satisfaction of multicultural children (Table 3).

**Table 3:** Moderating effect and moderated the mediating effect of resilience through acculturative stress on hardiness and life satisfaction.

**Variables**	***B***	***S.E.***	***t***	**LLCI**	**ULCI**
**Mediating variable model (Outcome variable: Acculturative stress)**					
Constant	9.414	3.675	2.561^*^	2.1665	16.6615
Hardiness	−0.273	0.104	−2.620^**^	−0.4785	−0.0675
**Dependent variable model (Outcome variable: Life satisfaction)**					
Constant	13.935	3.422	6.007^***^	9.3598	18.5099
Hardiness	0.015	0.086	0.220	−0.1155	0.1444
Acculturative stress	−0.076	0.038	−2.022^*^	−0.1505	−0.0019
Resilience	0.098	0.029	3.435^***^	0.0418	0.1545
Acculturative stress **×** Resilience	0.001	0.002	0.518	−0.0027	0.0047
**Conditional indirect effects of acculturative stress at resilience**					
Resilience	**Effect**	***S.E***.		**LLCI**	**ULCI**
M – 1SD	0.025	0.016		0.0009	0.0641
M	0.021	0.016		−0.0021	0.0588
M + 1SD	0.016	0.022		−0.0226	0.0686

* *p < 0.05*,

** *p < 0.01*,

*** *p < 0.001*.

*LLCI, lower level for confidence interval; ULCI, upper level for the confidence interval*.

The result shows that hardiness negatively influenced acculturative stress (*B* = −0.273, *p* < 0.001), but it did not significantly and directly influence life satisfaction in this model (*B* = 0.015, *p* = 0.826). Acculturative stress also negatively influenced life satisfaction (*B* = −0.076, *p* < 0.05). Thus, acculturative stress can completely mediates the relationship between hardiness and life satisfaction in this model.

Resilience positively influenced life satisfaction in this model (*B* = 0.098, *p* < 0.001), but, the interaction of acculturative stress and resilience did not significantly influence life satisfaction. This result indicates that resilience has no moderating effect on acculturative stress and life satisfaction.

Using bootstrapping, the conditional indirect effects of acculturative stress on resilience for life satisfaction (hardiness → acculturative stress → life satisfaction) were examined (Table 3). The results revealed that there was no zero between the upper and lower levels of the confidence interval for bootstrapping only at the level of −17.28 (mean centered, M – 1SD). Hayes (2015) stated that “If the confidence interval includes zero, then one cannot exclude no relationship between the moderator and the indirect effect from the realm of plausibility, meaning no definitive evidence of moderation of the mediation of X's effect on Y through M. But if the confidence interval does not include zero, this leads to the inference that the relationship between the indirect effect and the moderator is not zero—moderated mediation (p. 8)”. This result suggests that the mediating effect is significant only in the group with low resilience, i.e., people with a z-score of −1 or below. In other words, there was a moderated mediating effect in this model (Figure 1, Model 1).

### Verification of the Moderated Mediation Model for Life Expectation

This study also examined a moderating mediating effect of resilience through acculturative stress on the hardiness and life expectation of multicultural children (Table 4).

**Table 4:** Moderating effect and moderated the mediating effect of resilience through acculturative stress on hardiness and life expectation.

**Variables**	***B***	***S.E.***	***t***	**LLCI**	**ULCI**
**Mediating variable model (Outcome variable: Acculturative stress)**					
Constant	9.414	3.675	2.561^*^	2.1665	16.6615
Hardiness	−0.273	0.104	−2.620^**^	−0.4785	−0.0675
**Dependent variable model (Outcome variable: Life expectations)**					
Constant	20.597	2.100	9.807^***^	16.4551	24.7393
Hardiness	0.214	0.060	3.588^***^	0.0964	0.3316
Acculturative stress	−0.049	0.034	−1.448	−0.1167	0.0179
Resilience	0.186	0.026	7.199^***^	0.1352	0.2373
Acculturative stress **×** Resilience	0.006	0.002	3.243^**^	0.0022	0.0089
**Increase of** ***R***^**2**^ **with interaction**		***R*** ^**2**^		***F***	
		0.028		10.516^**^	
**Conditional effects of the focal predictor at values of the moderator (resilience)**					
**Resilience**	**Effect**	***S.E***.	***t***	**LLCI**	**ULCI**
M – 1SD	−0.145	0.043	−3.38^***^	−0.2296	−0.0605
M	−0.049	0.034	−1.45	−0.1167	0.0179
M + 1SD	0.046	0.047	0.98	−0.0468	0.1393
**Conditional indirect effects of acculturative stress by resilience**					
**Resilience**	**Effect**	***S.E.***		**LLCI**	**ULCI**
M – 1SD	0.040	0.022		0.0053	0.0870
M	0.014	0.010		−0.0023	0.0360
M + 1SD	−0.013	0.012		−0.0426	0.0039

* *p < 0.05*,

** *p < 0.01*,

*** *p < 0.001*.

*LLCI, lower level for confidence interval; ULCI, upper level for the confidence interval*.

The result revealed that hardiness negatively influenced acculturative stress (*B* = −0.273, *p* < 0.001), and positively and directly influenced life expectation in this model (*B* = 0.214, *p* < 0.001). However, acculturative stress did not significantly influence life expectation (*B* = −0.049, *p* = 0.149). This seems to be that acculturative stress cannot mediate hardiness and life expectation. Based on this result alone, acculturative stress does not seem to be able to mediate between hardiness and life expectation.

Resilience also positively influenced life satisfaction in this model (*B* = 0.186, *p* < 0.001). In addition, the interaction of acculturative stress and resilience significantly influenced life expectation (*B* = 0.006, *p* < 0.01), indicating that there is a moderating effect of resilience between acculturative stress and life expectation (Table 4). The change in *R*^2^ with the addition of interaction terms of acculturative stress and resilience was 0.028 and statistically significant. This result means that when the interaction term is added, this model will be able to account for 2.8% more variance in life expectation. We also checked whether this moderated mediating effect was moderated by gender using PROCESS Macro 3.5 Model 18, but it was not significant.

**Figure 2:**
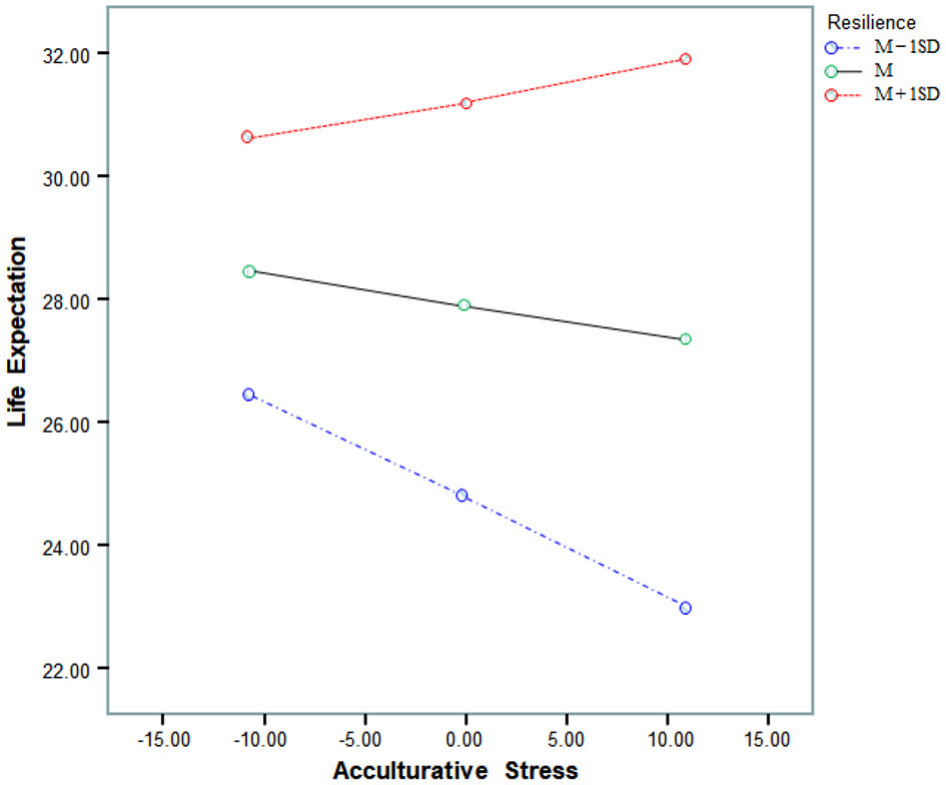
Interaction effect of acculturative stress and resilience on life expectation.

Figure 2 shows the interaction effect of acculturative stress and resilience for life expectation. In this model, people with less resilience (M – 1SD, z-score of −1 or below) were less likely to be hopeful about the future when levels of acculturative stress were high than they were low. In contrast, people with more resilience (M + 1SD, z-score of 1 or above) were more hopeful about the future when levels of acculturative stress was high than when they were low.

Using bootstrapping, the conditional indirect effects of acculturative stress on resilience for life expectation (hardiness → acculturative stress → life expectation) were examined (Table 4). Results indicated that there was no zero between the upper and lower levels of the confidence interval for bootstrapping only at the level of −17.28 (mean centered, M – 1SD). The mediating effect was significant only in the group with low resilience (people with a z-score of −1 or below) in this model. This results means that there was a moderated mediating effect of acculturative stress and resilience for life expectation (Figure 1, Model 2).

Table 5 presents the significance area analyzed with Johnson–Neyman's floodlight analysis method for the entire range of the moderating variable. This method shows where the moderating effects of the values of the moderator are significant.

**Table 5:** Significance area in the conditional effects of the focal predictor at values of the moderator (resilience).

**Value of resilience**	**β**	***S.E.***	***t***	**LLCI**	**ULCI**
−47.1294	−0.310	0.084	−3.681^***^	−0.4765	−0.1440
:	:	:	:	:	:
−10.7294	−0.109	0.037	−2.931^**^	−0.1820	−0.0356
−6.1794	−0.084	0.035	−2.409^*^	−0.1521	−0.0151
**−3.1948**	−0**.067**	**0.034**	–**1.972**	−0**.1342**	**0.0000**
−1.6294	−0.058	0.034	−1.721	−0.1254	0.0085
:	:	:	:	:	:
30.2206	0.118	0.065	1.830	−0.0094	0.2452
**33.7829**	**0.138**	**0.070**	**1.972**	−0**.0000**	**0.2752**
:	:	:	:	:	:
43.8706	0.193	0.085	2.270^*^	0.0254	0.3615

* *p < 0.05*,

** *p < 0.01*,

*** *p < 0.001*.

*LLCI, lower level for confidence interval; ULCI, upper level for the confidence interval*.

The influence of hardiness on life expectation through acculturative stress was significant in areas where resilience scores were less than −3.1948. In other words, acculturative stress mediates the relationship between hardiness and life expectation in areas where resilience scores were lower than −3.1948. This result means that multicultural children with high levels of hardiness experience less acculturative stress, which improves their expectation for their future life, only when the resilience is low. Conversely, low levels of hardiness makes multicultural children experience more acculturative stress, which reduces their expectation for their future life, which has no such mediating effect if they are highly resilient.

Interestingly, the mediating effect of acculturative stress on the relationship between hardiness and life expectation was only significant in participants with the resilience scores greater than 33.7829. As shown in Table 5, this effect was only found in cases where resilience was very high, and due to low levels of hardiness, multicultural children experience a lot of acculturative stress, but still had positive expectation for their futures.

## Discussion

This study explored the relationships among hardiness, acculturative stress, resilience, and the life satisfaction, and expectation of children from Korean multicultural families. It also examined the moderated mediating effects of resilience through acculturative stress on the relationship between hardiness and life satisfaction or expectation. The applications and implications of the study findings are discussed below.

The life satisfaction and life expectation of multicultural children shared only 4.1% variance (*r* = 0.202) in this study. In other words, the participants' expectations for the future are a different aspect of subjective well-being than their satisfaction with their present life for multicultural children. Kim et al. (2009) pointed out that even if life is unsatisfactory in youth, subjective well-being may not be considered low if they have the expectation for their future. The authors emphasized that expectation for the future is as important as life satisfaction is for the subjective well-being of youth. Therefore, it may be meaningful to consider the expectation as one of subjective well-being variables in this study.

As hypothesized in this study, the more acculturative stress is experienced by multicultural children, the lower their satisfaction or poorer their expectations for the future will be. This result suggests that acculturative stress of multicultural children could also reduce their well-being, similar to the effects of other kinds of stress (Ng et al., 2009). In particular, the expectation for the future is important during childhood and adolescence (Eckersley et al., 2005; Cho, 2011), and it has been found that acculturative stress is likely to lower the expectation for their future. In that sense, it is also highly suggestive that hardiness and resilience account for more variance of life expectation than life satisfaction for multicultural children because it was found that hardiness and resilience is likely to play an important role in promoting multicultural children's expectation for their future life and prevent the decrease of their life expectation.

In a moderated mediating model for life satisfaction, the direct path from hardiness to life satisfaction of multicultural children was not significant in this study. This result means that acculturative stress completely mediated the relationship between hardiness and life satisfaction for multicultural children. In other words, the low hardiness of multicultural children could decrease their life satisfaction if they experience acculturative stress. Since previous studies (Yoon et al., 2008; Bergnehr, 2018; Liang et al., 2020) and the present study has revealed that acculturative stress might be a determinant factor for life satisfaction or the well-being of multicultural children, teachers and counselors, who want to promote the well-being of multicultural students must work to decrease students' feelings of stress during the acculturation process.

The indirect path from hardiness to life satisfaction of multicultural children through acculturative stress is also moderated by resilience in this study. In this model, although resilience could not moderate the relationship between acculturative stress and life satisfaction of multicultural children, resilience moderated the indirect effect of acculturative stress on hardiness and life satisfaction. It means that for children with low resilience are more at risk of experiencing acculturative stress and become dissatisfied with their life, because of their low levels of hardiness. On the contrary, if multicultural children have high resilience, acculturative stress due to low hardiness will not likely decrease their life satisfaction. This result reiterates the explanation that hardiness is similar to temperament and a dispositional trait of personality (Kobasa et al., 1982; Bartone, 2007) and that resilience is a psychological ability or skill (de Terte and Stephens, 2014) that could be applied in crises or stressful events. It suggests that mental health professionals or counselors could be able to protect the well-being of multicultural children by working with them to develop resilience.

The moderating effect of resilience was more pronounced in the model for the life expectation of multicultural children than for their life satisfaction. The influence of acculturative stress on the life expectation of multicultural children was moderated by resilience in the model. It is conspicuous that this effect increases the accountability of the model about 2.8% for life expectation. Resilience could moderate the indirect effect of acculturative stress on the hardiness and life expectation of multicultural children. Analysis of the significance area for the conditional effects of the focal predictor for values of resilience revealed that the negative effect of acculturative stress due to low hardiness affects the life expectation of multicultural children even if their resilience is slightly lower than the average. In addition, the results indicated that very high levels of resilience could make multicultural children expect more from their future, even when there is acculturative stress due to low hardiness. This finding suggests that resilience plays a very important role in multicultural children's expectation of their future in stressful situations for multicultural children. Therefore, research is needed that can be used educationally and clinically is needed. Lyubomirsky and Della Porta (2010) introduced cognitive and behavioral interventions to increase resilience, which needs to be applied in multicultural children with low resilience.

This study showed that hardiness and resilience can maintain and promote multicultural children's life satisfaction and expectation during the acculturation process, and provides academically, educationally, and clinically useful information, which are necessary as the world becomes more global and multicultural. Despite the implications, there are some limitations to be considered. First, the sample of this study is not necessarily representative of Korean multicultural children, or other international multicultural children, because data were collected from certain areas of Korea and their multicultural families were formed because of their mothers, who were migrant women. Second, this study specifically targeted children in early adolescence, and the scales used in this study were not specialized for this age group. Therefore, further studies are needed to re-verify the results of this study after more age specific measures are developed. Third, because no conclusion can be reached from a single study, the role of hardiness, acculturative stress, and resilience in life satisfaction or expectation of multicultural children should be explored further in the future. For example, it is also necessary to ascertain whether the findings of this study vary by gender and further research on various factors that threaten the mental health and well-being of children with low resilience are needed. Finally, we discussed the cause-and-effect relationship based on the results of previous studies, but causation cannot be completely concluded on the basis of results.

## Conclusion

This study investigated the relationships among hardiness, acculturative stress, and life satisfaction or expectation of children from Korean multicultural families, focusing on the mediating effects of acculturative stress on hardiness and life satisfaction or expectation moderated by resilience. No moderating of resilience was found on acculturative stress and life satisfaction, and acculturative stress completely mediated the relationship between hardiness and life satisfaction in multicultural children. A moderated mediating effect was found for life expectation, and a mediating effect of acculturative stress on hardiness stress and life expectation was only significant in groups with low resilience and with very high resilience. Although study had its limitations of the study exist, it is expected that the findings would provide valuable knowledge for future research and useful information for education and counseling professionals who wish to improve the well-being of multicultural children.

## Data Availability Statement

Any queries regarding the data used in this study may be directed to the corresponding author.

## Ethics Statement

The studies involving human participants were reviewed and approved by Institutional Review Board (IRB) of Sahmyook University (2-1040781-A-N-012020015HR). Written informed consent to participate in this study was provided by the participants' legal guardian/next of kin.

## Author Contributions

B-HK collected data and conducted the literature review. K-HS analyzed/interpreted the data and wrote the final manuscript. Both authors designed the study and agreed to be responsible for all aspects of this study.

## Funding

This study was supported by the fund of the Sahmyook University.

## Conflict of Interest

The authors declare that the research was conducted in the absence of any commercial or financial relationships that could be construed as a potential conflict of interest.

## Publisher's Note

All claims expressed in this article are solely those of the authors and do not necessarily represent those of their affiliated organizations, or those of the publisher, the editors and the reviewers. Any product that may be evaluated in this article, or claim that may be made by its manufacturer, is not guaranteed or endorsed by the publisher.

## Abbreviations

ADHD: attention deficit hyperactivity disorder
K-SAFE-C: Korean version of societal, attitudinal, familial, and environmental acculturative stress scale for children
LEMS: life satisfaction expectancy motivation scale
SPSS: statistical package for social sciences
SWLS: satisfaction with life scale.
